# Electronic origin of antimicrobial activity owing to surface effect

**DOI:** 10.1038/s41598-018-37645-w

**Published:** 2019-01-31

**Authors:** Naoki Miyazawa, Susumu Sakakibara, Masataka Hakamada, Mamoru Mabuchi

**Affiliations:** Graduate School of Energy Science, Kyoto University, Yoshidahonmachi, Sakyo, Kyoto, 606-8501 Japan

## Abstract

Nanomaterials have displayed promising potential as antimicrobial materials. However, the antimicrobial mechanism owing to surface effects, where the emission of harmful substances such as metallic ions and reactive oxygen species is not required, is still poorly understood. It is important to figure out relationship between the physical properties and antimicrobial activity based on deep understanding of antimicrobial mechanism for their safe and effective applications. Here, we show that the work function is representative of the surface effect leading to antimicrobial activity, which originates from the electronic states of the surface. We investigated the antimicrobial activity and the work function of nanoporous Au-Pt and Au without the emission of Ag ion, and found that there was a positive correlation between them. In addition, we performed a first-principles calculation and molecular dynamics simulation to analyze the electronic states of the Au surface and the cell wall. These demonstrated that positive correlation was owing to peculiar electronic states at the Au surface, namely, the spilling out phenomenon of electrons. Our finding will contribute to advance the understanding of biological phenomena from a physical view.

## Introduction

The surface effect owing to peculiar electronic states at the surface is one of the most important properties of metallic nanomaterials. However, there is much room for understanding the surface effect on large organic matters such as bacteria, whereas there are many studies on small molecules such as amino acids^1,2^. Actually, the remarkable antimicrobial activity (AA) of metallic nanomaterials such as Au nanoparticles is not directly caused by the surface effect because the AA results from the emission of harmful substances such as metallic ions and reactive oxygen species (ROS)^3–5^. There are other kinds of antimicrobial metallic nanomaterials that kill bacteria through incorporation into the cytoplasm and deterioration of cytoplasmic proteins without releasing ROS and metallic ions^6–8^. Understanding of the origins of AA without the release of harmful substances is required for the safe application of antimicrobial nanomaterials.

Nanoporous Au (npAu) shows high catalytic activities on organic molecules such as the oxidation of carbon monoxide^9^ and methanol^10^. The catalytic activities of npAu originate from the peculiar electronic states on their surfaces. Recently, higher AA of npAu compared with that of flat Au (fAu) was reported^11^. The AA of npAu does not include the emission of harmful substances such as metallic ions and reactive oxygen species. In addition, the npAu did not exhibit AA in a high relative humidity of 90%, indicating that the direct contact of the bacteria on the npAu surface was necessary for the high AA. NpAu specimens cannot pass through the cell wall of bacteria because of their bulky dimensions, with typical macroscopic lengths larger than one millimeter. These facts suggest that the high AA of npAu can be attributed to the surface effect. Because of the inertness of gold, organic molecules are adsorbed on Au surfaces through physisorption owing to Coulombic or van der Waals interactions and not owing to chemical (covalent/ionic) binding. Therefore, the surface effect of npAu was related to the Coulombic or van der Waals interactions between the bacterial cell wall and the npAu surface. However, the physical origins of the surface effects in npAu are still not sufficiently understood.

A recent study showed that the cell wall of bacteria was negatively hyperpolarized after contact with the npAu surface and the hyperpolarized cell wall caused the structural change of ion channels, which led to the AA of npAu^12^. Electrons spill out on a metallic surface and an electric double layer is formed, resulting in a positive metallic surface^13,14^. Therefore, the nature of peculiar electronic states at the surface, which is responsible for the AA, is related to the spilling out of electrons. Therefore, it is worth estimating the intensity of the spilling out of electrons for deep understanding of the origin of the AA. The molecular dipole is closely connected with the work function (WF)^15,16^. Also, the interface dipole moment is generated during the adsorption of organic molecules^17,18^. Therefore, the hyperpolarization of the cell wall of bacteria, which is induced by npAu, is suggested to have a correlation with the WF of npAu. In the present work, the effectiveness of WF as an indicator of AA was demonstrated by investigating Au specimens with different WFs. There are some methods to vary the WF of metallic surfaces: adsorption of molecules^17,19^, modification of self-assembled monolayers (SAMs)^16,20^, alloying^21,22^ and lattice distortion^23,24^. The adsorption of molecules and SAM modification were not appropriate in the present work because the effect of the adsorbates themselves on the AA cannot be neglected. Therefore, alloying was appropriate for varying the WF of npAu. We chose platinum (Pt) as an alloying element because ion elution of Pt can be neglected owing to its nobleness (or low ionization tendency).

In the present work, the AAs of nanoporous Au-Pt (npAu-Pt_0.5_ and npAu-Pt_0.1_, see Methods for details), npAu and fAu specimens were investigated on *E. coli*. Also, their WFs were calculated from ultraviolet photoelectron spectrometry (UPS) measurements. Furthermore, a first-principles calculation and molecular dynamics (MD) simulations were performed to analyze the electronic states of the surface and the cell wall. To the best of our knowledge, the present paper is the first work showing that the WF can be representative of the surface effect on the biological phenomenon of AA.

## Results and Discussion

**Figure 1:**
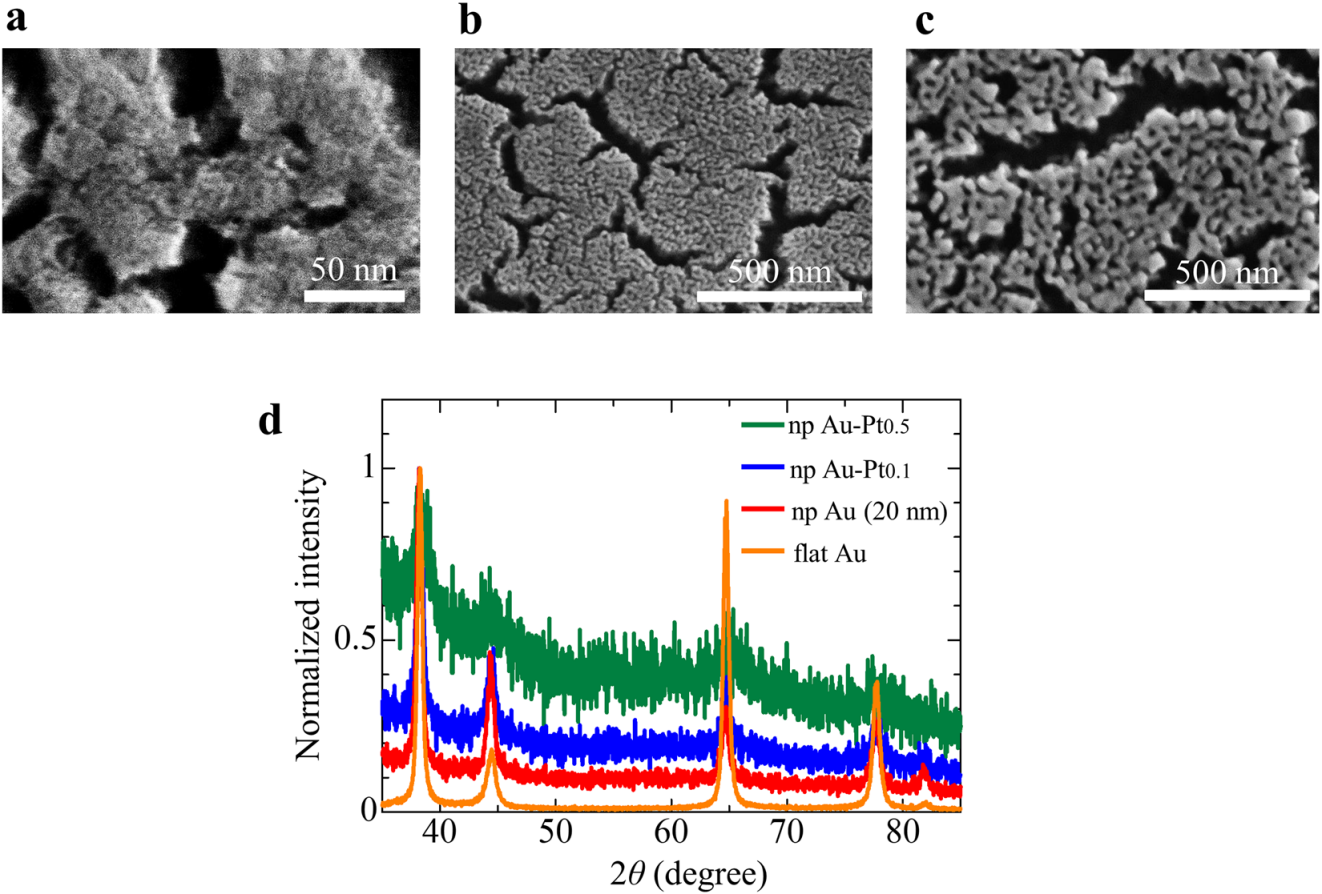
Nanoporous Au-Pt and Au fabricated by dealloying. (**a**–**c)** SEM images of npAu-Pt_0.5_, npAu-Pt_0.1_ and npAu (20 nm). (**d**) XRD measurements of npAu-Pt_0.5_, npAu-Pt_0.1_, npAu (20 nm) and flat Au (fAu), where green, blue, red and orange lines show XRD of npAu-Pt_0.5_, npAu-Pt_0.1_, npAu (20 nm) and flat Au (fAu), respectively.

The nanoporous Au-Pt and Au are shown in Fig. 1. The average ligament and pore size were 5 and less than 2 nm for npAu-Pt_0.5_, 15 and approximately 10 nm for npAu-Pt_0.1_, and 34 and approximately 20 nm for npAu. The ligament and pore sizes of npAu-Pt were lower than those of npAu^25^. The smaller pore structure of the npAu-Pt alloy was attributed to the lower surface diffusion of Pt^26^. Alloying of npAu with Pt reduced the lattice constant (Fig. 1d). The reduction in the lattice constant by the Pt addition almost corresponded to Vegard’s law.

Residual Ag in npAu-Pt and npAu may affect the AA because the Ag ion is a strong killer of bacteria^3^. The Ag concentration was 16.75 at.% for npAu-Pt_0.5_, 7.23 at.% for npAu-Pt_0.1_ and 0.85 at.% for npAu (Supplemental Data Table 1). However, the Ag concentration in the culture medium after the AA tests was less than the detection limit of the inductively coupled plasma (ICP) atomic emission spectrophotometry measurements. Therefore, the effect of residual Ag on the AA was low enough to be ignored.

**Figure 2:**
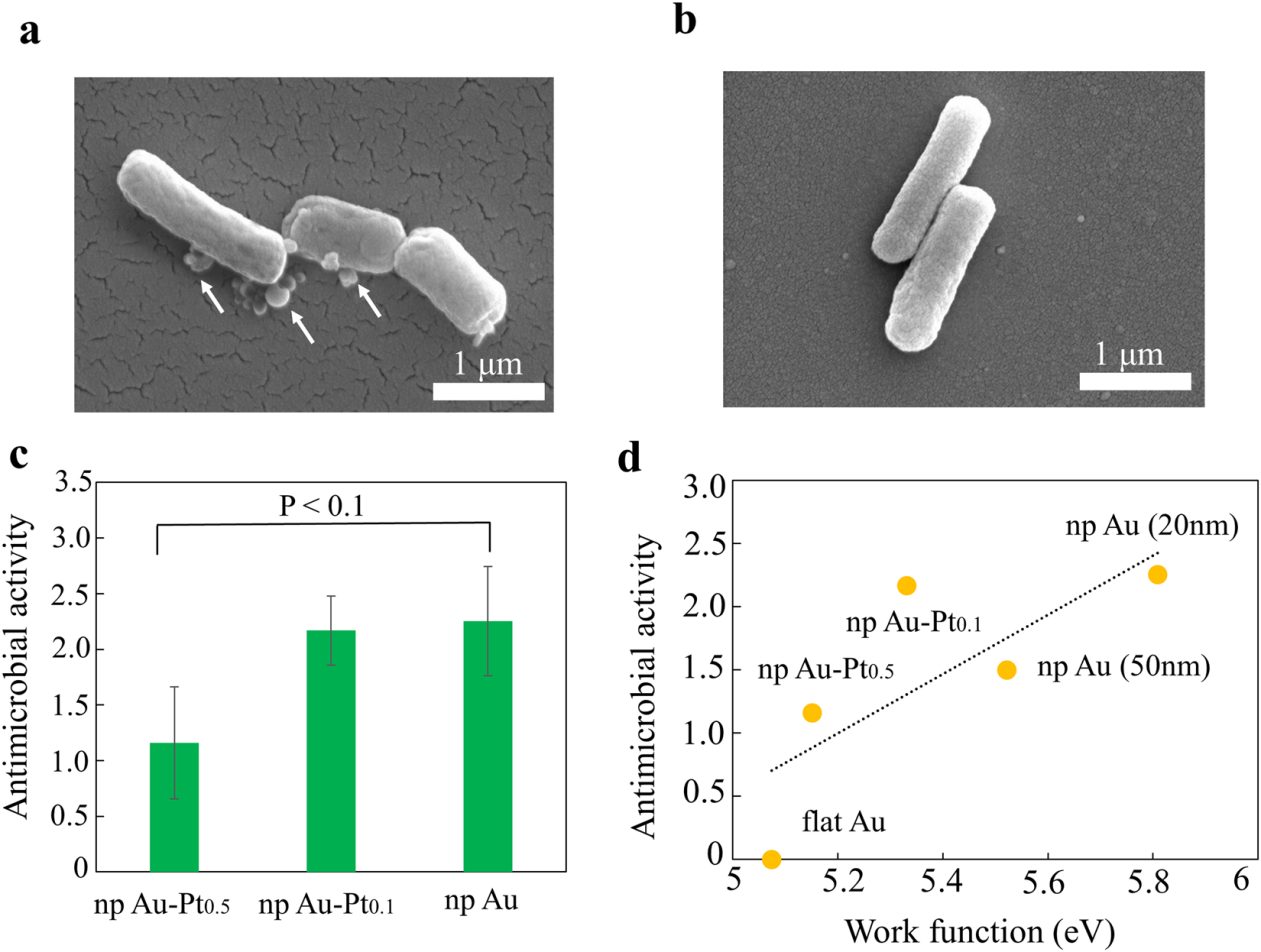
Results of antimicrobial activity tests. (**a**) SEM image of *E. coli* cultured on npAu-Pt_0.5_. *E. coli* cultured on npAu-Pt_0.5_ was dead and its cell membrane was broken (cytoplasm drained out of bacterial body, as indicated by arrows). (**b**) SEM image of *E. coli* cultured on flat Au (fAu). *E. coli* cultured on fAu was not dead. (**c**) Antimicrobial activities (AA) of npAu-Pt and npAu. (**d**) Relationship between AA and work function (WF). In the figure, npAu (20 nm) is for npAu with a pore size of 20 nm and npAu (50 nm) is for npAu with a pore size of 50 nm. The AA of npAu (50 nm) is the one obtained in the previous study^11^.

Figure 2 shows results of the antimicrobial tests. A large number of *E. coli* cultured on the npAu-Pt_0.5_ were dead, whereas only a few *E. coli* cultured on the fAu were dead in the AA tests. It appeared that the cell membrane was broken for *E. coli* cultured on the npAu-Pt_0.5_ (Fig. 2a,b). This corresponded to that for *E. coli* cultured on npAu^11^, suggesting that the AA mechanism for npAu-Pt was the same as that for npAu. The AAs were 1.2 ± 0.5, 2.2 ± 0.3 and 2.3 ± 0.5 for npAu-Pt_0.5_, npAu-Pt_0.1_ and npAu, respectively (Fig. 2c). Differences between npAu-Pt_0.5_ and npAu were considered statistically significant at a P-value < 0.1. Alloying with Pt reduced the AA of npAu. The reduced AA by alloying with Pt was not due to residual Ag because the residual Ag contents for the npAu-Pt were larger than that for the npAu. The smaller pore and ligand size enhanced the AA of npAu^11^. Hence, the finer pore structure of the npAu-Pt was not responsible for the reduced AA after alloying with Pt.

The WF was measured from the UPS spectrum (Supplemental Fig. 1). The measured work function is listed in Table 1. The WF of fAu agreed with the value measured from the Fowler plot^27^. The WFs of npAu-Pt and npAu were larger than that of fAu, indicating that the surface effect enhanced the spilling out of electrons at the surfaces. Noted that there was a positive correlation between the AA and the WF, in which the coefficient of correlation was 0.73 (Fig. 2d). This demonstrated that the WF was representative of the intensity of the surface effect that leads to AA. The two series of npAu-Pt showed lower AA than the npAu with a pore size of 20 nm, despite the smaller pore size of npAu-Pt. It is therefore suggested that alloying with Pt changed the electronic states, resulting in reduced AA.

**Table 1:** Work function measured from the UPS spectrum.

Specimens	Work function (eV)
npAu-Pt_0.5_	5.15
npAu-Pt_0.1_	5.33
npAu(20 nm)	5.81
npAu(50 nm)	5.52
fAu	5.07

We performed first-principles calculations to investigate the origins of the variation in the electronic states after alloying with Pt. In the calculation, the WF was defined as the energy difference between the electrostatic potential at the middle of the vacuum region and the Fermi energy^14^. The order of calculated WF was WF(npAu) > WF(npAu-Pt) > WF(fAu) (Supplemental Fig. 2). This trend agreed with the experimental one. Nanoporous metals have large lattice strains of up to 10% at the surfaces, because the nanosized ligaments have high positive and negative curvatures^26,28^. Therefore, a 5% compressive strain was loaded in the simulation models of npAu and npAu-Pt; the larger WFs for npAu-Pt and npAu can be explained from the compressive strain^23,24^.

**Figure 3:**
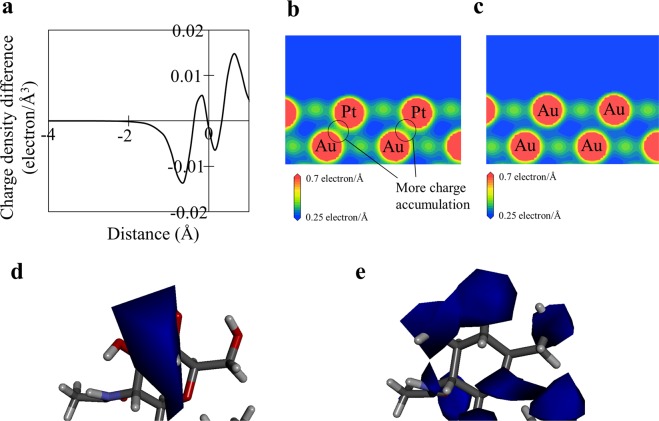
Results of first-principles calculation and molecular dynamics simulation. (**a**) Electron charge difference between npAu-Pt and npAu (=*ρ*_npAu-Pt_ − *ρ*_npAu_, in which *ρ*_npAu_ and *ρ*_npAu-Pt_ are the electron density for npAu and for npAu-Pt, respectively) of the (100) plane. The (100) plane containing no Pt atoms was investigated for npAu-Pt. The horizontal axis shows the distance from the center of the first layer Au atom, and the positive (negative) value indicates the bulk (vacuum) region. (**b**,**c**) Charge density of the (121) plane for npAu-Pt and for npAu. The (121) plane containing three Pt atoms was investigated for npAu-Pt. (**d**,**e**) Electrostatic potential of peptidoglycans located on npAu-Pt and on npAu, where gray, white and red atoms are carbon, hydrogen and oxygen atoms, respectively, and the blue region show the isosurface of electrostatic potential. The isosurface value is negative value of −6kT.

The WF mainly results from the spilling out of electrons and electric double layer formation at the metallic surface^13,14^. Hence, the variation in WF by alloying with Pt can be investigated by the electron charge difference between npAu-Pt and npAu (=*ρ*_npAu-Pt_ − *ρ*_npAu_, in which *ρ*_npAu_ and *ρ*_npAu-Pt_ are the electron density of npAu and npAu-Pt, respectively). The first-principle calculations were performed to investigate the electron charge difference. The result is shown in Fig. 3a. The electron charge difference was negative in the vacuum region except for the Au atomic radius. This means that alloying with Pt reduces the spilling out of electrons. The charge densities of the surfaces are shown in Fig. 3b,c. More charge was accumulated between the first and the second layer of npAu-Pt than of npAu because the charge transfer occurred from Pt to Au atoms for npAu-Pt. On the other hand, the chemical (covalent) bonding between Au and Pt atoms may also affect the variation in WF^29^. However, such chemical bonding was not generated between Au and Pt atoms in npAu-Pt (Supplemental Fig. 3). Therefore, it is suggested that the reduced WF by alloying with Pt was mainly related to the charge transfer from Pt to Au atoms. Another factor affecting the WF is the geometric effect^22^. There are a large number of atomic steps at the surfaces of npAu^30^. The density of atomic steps may be higher for npAu-Pt than for npAu because of the smaller pore structure of npAu-Pt. The higher density of atomic steps can reduce the WF owing to the Smoluchowski mechanism^13^. Therefore, the lower WF of npAu-Pt may be partly related to the higher density of atomic steps.

The electrostatic potential of peptidoglycans interacting with npAu-Pt and with npAu is shown in Fig. 3d,e. The peptidoglycans interacting with npAu-Pt as well as with npAu were negatively hyperpolarized, but the intensity of hyperpolarization of the peptidoglycan with npAu-Pt was weaker than that with npAu. This is because alloying with Pt reduced the spilling out of electrons. Therefore, the reduced spilling out of electrons owing to alloying with Pt weakened the hyperpolarization of bacterial cell walls. This resulted in a reduction in AA by alloying npAu with Pt.

A possible approach for further elucidation of the whole mechanism and phenomena is the analyses of gene expression in *E. coli* such as real-time PCR and microarray analysis^11^. The approach may make it possible to clearly differentiate the antimicrobial mechanism of nanoporous noble metals from that of noble metal nanoparticles.

## Conclusions

The antimicrobial activity and the work function of nanoporous Au-Pt, nanoporous Au and flat Au were investigated. As a result, there was a positive correlation between them. Thus, the work function was representative of the surface effect leading to the antimicrobial activity. A first-principles calculation and molecular dynamics simulation showed that the positive correlation is owing to peculiar electronic states at the Au surface, namely, the spilling out phenomenon of electrons.

## Methods

### Preparation of npAu and npAu-Pt

A 100-nm-thick pure gold film (>99.9 mass%) was sputtered on a 50 × 50 × 1.2 mm glass substrate. 150-nm-thick Au_0.3_Ag_0.7_, (Au_0.5_Pt_0.5_)_25_Ag_75_, and (Au_0.9_Pt_0.1_)_25_Ag_75_ films were then sputtered on the pure gold film. Nanoporous Au and Au-Pt specimens were fabricated by dealloying (free corrosion) of these films at 253 K for 24 h in 69 mass% HNO_3_. Also, a nanoporous Au specimen with a larger pore of 50 nm was fabricated by dealloying at 298 K. Nanoporous Au and Au-Pt made of Au_0.3_Ag_0.7_, (Au_0.5_Pt_0.5_)_25_Ag_75_ and (Au_0.9_Pt_0.1_)_25_Ag_75_ were denominated “npAu”, “npAu-Pt_0.5_” and “npAu-Pt_0.1_”, respectively. Immediately after dealloying, the specimens were thoroughly rinsed more than 10 times with pure water. A flat Au (fAu) specimen, which was fabricated by sputtering of pure gold, was used as a reference inert substrate. The microstructures of npAu-Pt and npAu specimens were observed by scanning electron microscopy (SEM; SU-6600 by Hitachi High-Technologies Corporation). The average ligament sizes were calculated by measuring the diameter of >50 ligament, while the average pore sizes were calculated by averaging >50 spacing between ligaments, except for npAu-Pt_0.5_ sample whose ligaments and pores were too small to observe clearly by SEM. X-ray diffraction (XRD; X’Pert Pro by PANalytical) measurements were performed on the npAu-Pt, npAu and fAu specimens. Their chemical compositions were investigated by energy-dispersive X-ray (EDX; XFlash 5010, Bruker AXS, Germany) spectroscopy.

### Bacterial strain

Type strains of *E. coli* (K-12, NBRC 3301) were supplied by the National Institute of Technology and Evaluation (Tokyo, Japan). We incubated the bacteria in Luria-Bertani (LB) medium at 308 K for 44 h before treating them in antimicrobial tests. Casein-peptone glucose yeast extract LB (Wako Pure Chemical Industries Ltd., Osaka, Japan) was used for the incubation.

### Tests of antimicrobial activity (AA)

The antimicrobial properties of npAu-Pt, npAu and fAu were investigated mainly according to the Japanese Industrial Standard (JIS) “Antibacterial products-Test for antibacterial activity and efficacy”^31^. First, one quantity of platinum loop of bacteria incubated in the medium was removed from the colony and placed in 5 mL of 1/500 nutrient broth, followed by vortex mixing. Second, 400 μL of the bacterial suspension was dropped onto the samples and then a 40 × 40 mm PE film covered the bacterial suspension. In this way, bacterial suspensions were incubated on the specimen for 24 h in humidity-controlled incubators at 308 K and at a relative humidity (RH) of 50%. The RH is 90% in JIS; however, the AAs for fAu and npAu were almost zero at an RH of 90%^11^. Therefore, the AA tests were carried out at the intermediate RH of 50%. Third, the incubated bacteria were recovered using 10 mL of Soybean Casein Digest Broth with Lectithin & Polysorbate 80 (SCDLP) medium and diluted 10-fold in phosphate-buffered saline (PBS). The diluted PBS was mixed in LB medium to make a 10-fold dilution series of LB pour plates. These were then incubated at 308 K for 48 h. The number of colonies in the LB pour plates was then counted. Viable bacteria counts (VBCs) were statistically analyzed by the one-way analysis of variance followed by a post-hoc test. The AA was given by

1 $${\rm{AA}}={\mathrm{log}}_{10}({N}_{0}/N),$$

in which *N*_0_ is the viable bacteria count for fAu (as a control sample) and *N* is the viable bacteria count for npAu-Pt or npAu. The mean value of AA was obtained from 5 repeated tests. All results are expressed as mean ± standard deviation.

### Inductively coupled plasma (ICP) atomic emission spectrophotometry measurements

The culturing solution was suspended on the npAu-Pt substrate for 24 h, and the sample was then analyzed using ICP atomic emission spectroscopy. The concentrations of silver, gold, and platinum ions in the culturing solutions were found to be < 0.05 ppm of the apparatus detection limit. At least approximately 1 ppm is necessary for realizing the antimicrobial properties of Ag ions^4,32–34^. Therefore, the effect of Ag ion dissolution on AA could be ignored.

### Ultraviolet photoelectron spectrometery (UPS) measurements

WFs of npAu-Pt, npAu and fAu were measured with a PHI 5000 VersaProbe II Scanning ECSA Microprobe system (ULVAC-PHI, Chigasaki, Japan). A windowless helium discharge light source that provided He1 emission at 21.22 eV was used. The diameter of a vacuum-ultraviolet (VUV) light beam was 5 mm and the incident angle was 45°. The samples were biased at −5 V dc to drive low-energy secondary electrons into the detector to prevent signal cut-off owing to the detector. The work function (WF) ${\rm{\Phi }}$, can be given by

2 $${E}_{{\rm{fermi}}}-{E}_{{\rm{cutoff}}}={\rm{\Phi }}-h\nu ,\,$$

in which *E*_fermi_ is the binding energy of the electron at fermi level, *E*_cutoff_ is the energy of the low-energy secondary electron, and *hv* is the photon energy (21.22 eV). Before the measurements, the surfaces were cleaned by removing organic molecules using gas cluster ion beam (GCIB) of Ar emission for 5 minutes.

### First-principles calculations of Au surfaces

We performed first-principles calculations for geometry optimization calculation of Au surface models by using the Cambridge Serial Total Energy Package (CASTEP)^35^, in which a plane-wave basis set was used to calculate the electronic properties based on density functional theory (DFT)^36,37^. The Perdew-Burke-Ernzerhof functional (PBE) version of the generalized gradient approximation^38^ was used to represent exchange and correlation interactions within the DFT. Ultrasoft pseudopotentials^39^ were used for all elements in the calculations. The cutoff energy was set to 320 eV and the Brillouin zone was sampled using 5 × 5 × 1 Monkhorst-Pack k-point meshes in all calculations^40^. Periodic boundary conditions were applied in the x, y, and z directions for all of the calculations.

A slab geometry with 4 atomic layers of 4 × 4 and a vacuum layer of 30 Å was used to model npAu-Pt, npAu and fAu surfaces (Supplemental Fig. 4). In the models, Ag atoms were not considered because effects of Ag atoms on the AA were ignorable. The atoms at the top three layers were relaxed to their equilibrium positions and the atoms at the bottom layer were frozen at their bulk positions in the models. Nanoporous metals have large lattice strains of up to 10% at the surfaces^26,28^. A previous study^12^ showed that a cell wall was hyperpolarized when the cell wall was adsorbed on the (111) surface of npAu with 5% compressive lattice strain. Thus, the Au (111) surface with 5% compressive lattice strain was used as the npAu model in the present study. To create a npAu-Pt model, three Au atoms of the first layer in the npAu model were substituted by Pt atoms. The Pt concentration in the Au-Pt model almost corresponded to the experimental one, which was detected by XPS for npAu-Pt_0.5_. The WF was calculated with these surface models, in which the WF was defined as the energy difference between the electrostatic potential at the middle of the vacuum region and the Fermi energy^14^.

### Molecular dynamics simulation and first-principles calculations of hyperpolarization of peptidoglycan

The hyperpolarization of peptidoglycan interacting with npAu-Pt, npAu or fAu was calculated by first-principles calculations and molecular dynamics (MD) simulations with the same methods used in a previous study^12^. A scaffold model of peptidoglycan was constructed. The peptidoglycan was immersed in a spherical water solvation, where the center of water solvent was positioned at the mass center of peptidoglycan and the diameter of the spherical solvent water was 50.0 nm. Counter ions of 43 Na^+^ and 43 Cl^−^ were added to neutralize the system. The system was energy-minimized using the steepest decent algorism (200,000 steps) and the conjugate gradient algorism (100,000 steps). MD simulations were performed with a time step of 2.0 fs. The system was gradually heated from 5 to 300 K for 4 ps to activate thermal motion in the system. The system was equilibrated for 1 ns to obtain a stable structure of peptidoglycan with a constant number of particles, volume and temperature (NVT). Finally, the 10 ns NVT simulations were performed.

An interaction between MurNAc, which is a part of peptidoglycan, and the Au surface was calculated by first-principles calculations. A $4\sqrt{3}\times 3\sqrt{3}$ unit cell, which consisted of four Au layers, with a lattice strain of −5% was used as a npAu surface model (Supplemental Fig. 5). A vacuum gap of 15 Å was added to create the surface. For the npAu-Pt model, 12 Au atoms of the surface layers were substituted by Pt atoms, in which the replaced positions were the same as those in Supplemental Fig. 4 (Supplemental Fig. 5). The geometry optimization calculations were performed on the Au surface models by first-principles calculations using the Dmol3 code^41,42^. In the DMol3 method, the physical wave functions were expanded in terms of the accurate numerical basis sets. The exchange-correlation energies were treated according to the generalized gradient approximation (GGA) with the Perdew-Wang 1991 (PW91) approximation^43^ to deal with the core (DNP). The ultrasoft pseudopotentials^39^ represented in reciprocal space were used for all elements in the calculations. Optical Bloch equation (OBE) calculations were used to set the van der Waals interactions into calculations. A Fermi smearing of 0.005 hartree (1 hartree = 27.2114 eV) was adopted. A Brillouin zone of 2 × 2 × 1 using a Monkhorst-Pack k-point mesh^40^ was used. The bottom layer of the cell was frozen during geometry optimization calculations. MurNAc was positioned to be the atop site^12^.

After the geometry optimizations of a MurNAc molecule located on the atop site of the Au surface model, a MurNAc molecule was put back at the same position in the original peptidoglycan model, and 1 ns MD simulations were performed again, in which the atomic positions of the MurNAc molecule were fixed during the calculations. Then, electrostatic potentials of the obtained peptidoglycan were calculated by solving the Poisson Boltzmann equation using the finite difference method implemented in the Delphi program^44,45^. The values of the atomic radii and partial atomic charges were taken from the CHARMM parameter set. The peptidoglycan was divided into a three-dimensional cubical grid and the electrostatic potential at each grid point was computed.

## Supplementary information

Supplemental information
