# Supplementary material for: Electronic origin of antimicrobial activity owing to surface effect
Source: Sci Rep. 2019 Jan 31;9:1091. doi: 10.1038/s41598-018-37645-w (PMC6355919; doi:10.1038/s41598-018-37645-w)
Supplement: Supplementary file 1 — Supplemental information [file 41598_2018_37645_MOESM1_ESM.docx]

**Suppplemental Information for Electronic origin of antimicrobial activity owing to surface effect**

Naoki Miyazawa*, Susumu Sakakibara, Masataka Hakamada and Mamoru Mabuchi

Graduate School of Energy Science, Kyoto University,

Yoshidahonmachi, Sakyo, Kyoto 606-8501, Japan


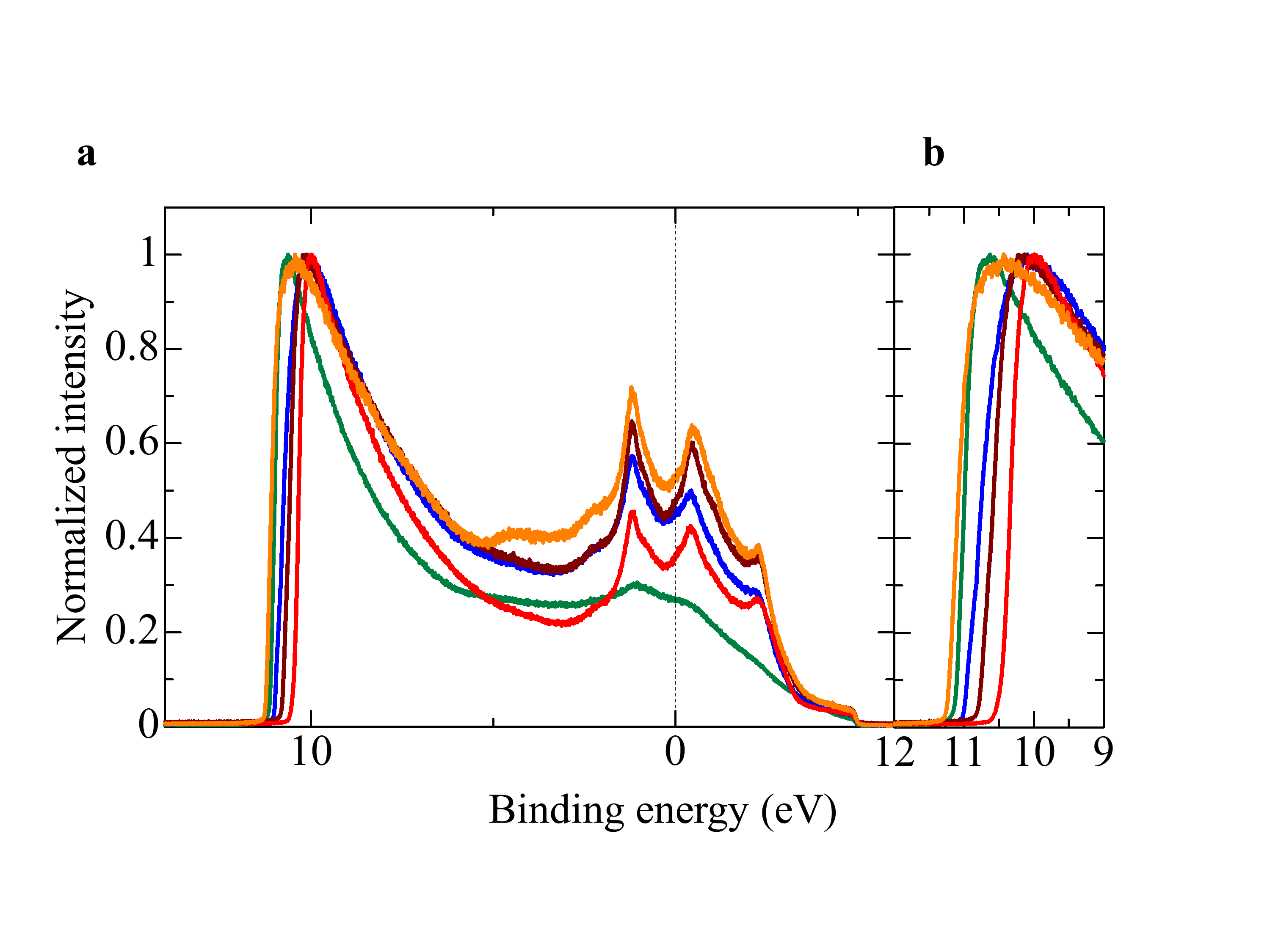


**Supplemental Figure 1. Spectrums obtained by UPS measurements.** **a**, Whole spectrum. **b**, Initial part of the spectrum in which green, blue, brown, red and orange lines are the spectra of npAu-Pt_0.5_, npAu-Pt_0.1_, npAu (50 nm), npAu (20 nm) and flat Au, respectively. The work function is calculated from the energy at the flexion point of the spectrum.


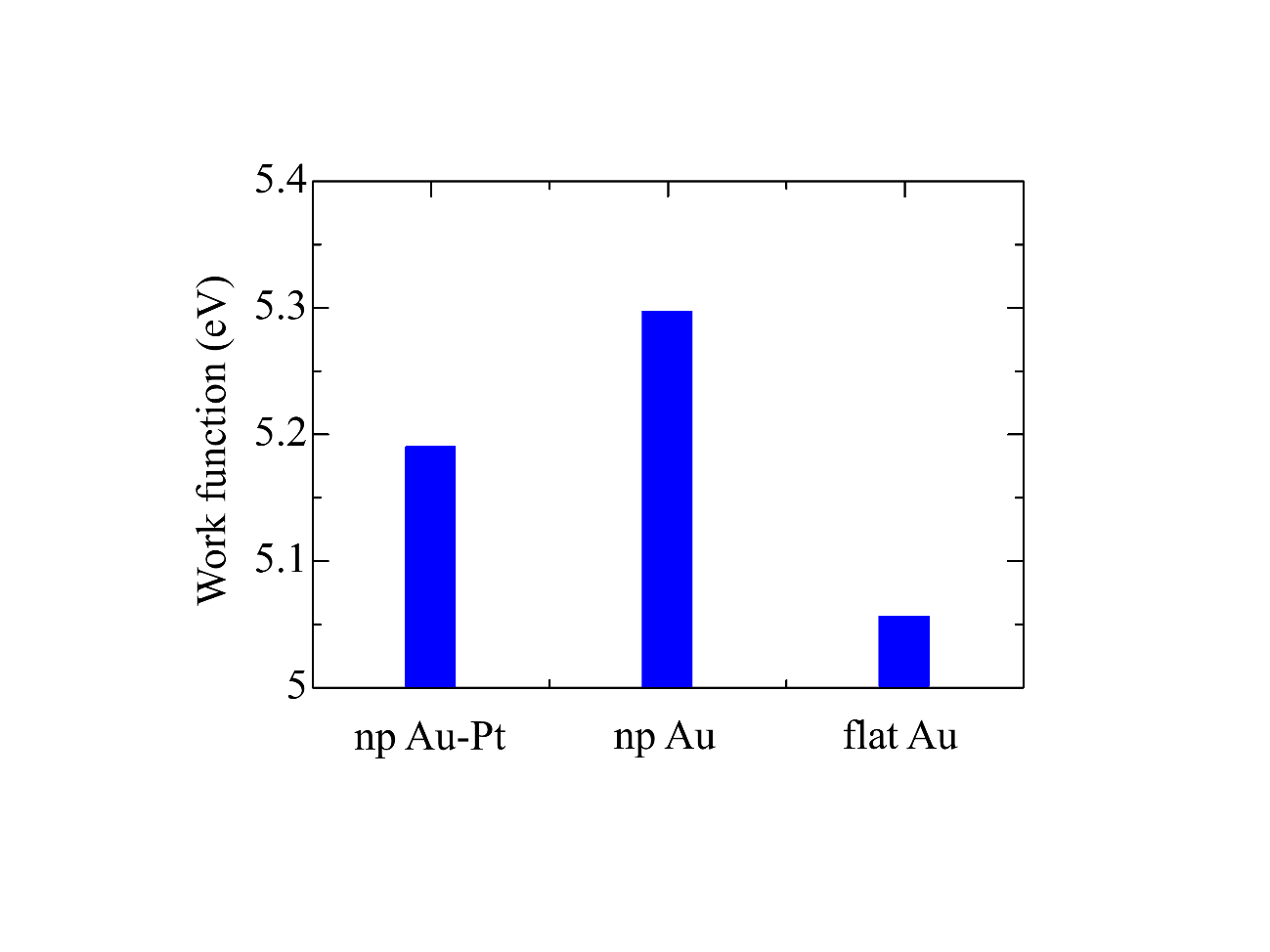


**Supplemental Figure 2. Work function obtained by first-principles calculation.** The work function (WF) is defined as the energy difference between the electrostatic potential at the middle of the vacuum region and the Fermi energy. The order of calculated WF is WF(npAu) > WF(npAu-Pt) > WF(fAu).


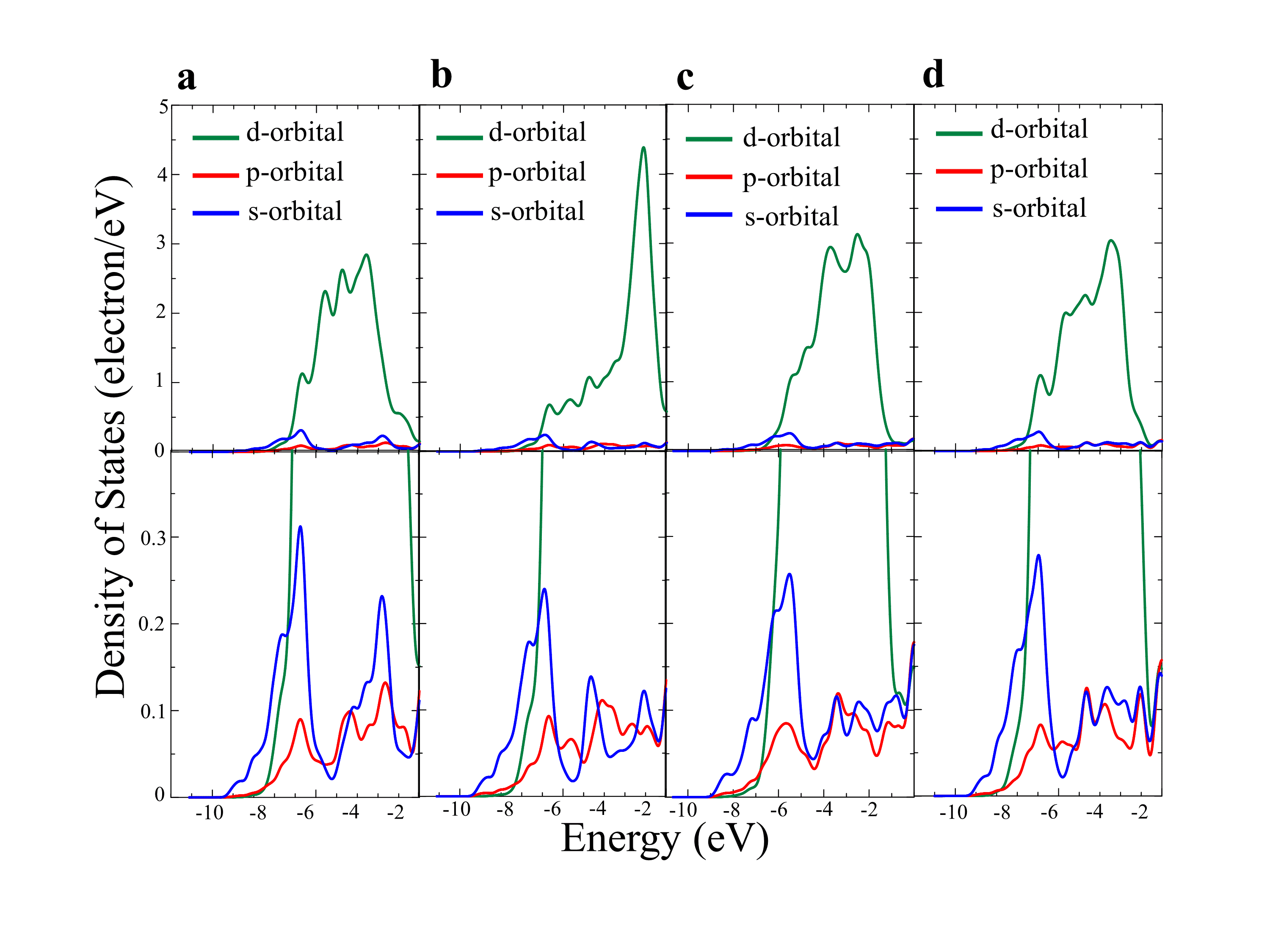


**Supplemental Figure 3. Density of electronic states (DOS).** **a**, DOS of Au atom in npAu-Pt. **b**, DOS of Pt atom in npAu-Pt. **c**, DOS of Au atom in npAu. **d**, DOS of Au atom in flat Au. The green, blue and red lines show s-, p- and d-orbitals, respectively. The upper panels focus on DOS of d-orbitals and the lower panels on DOS of s- and p-orbitals. Chemical (covalent) bonding is not generated between Au and Pt atoms in npAu-Pt.


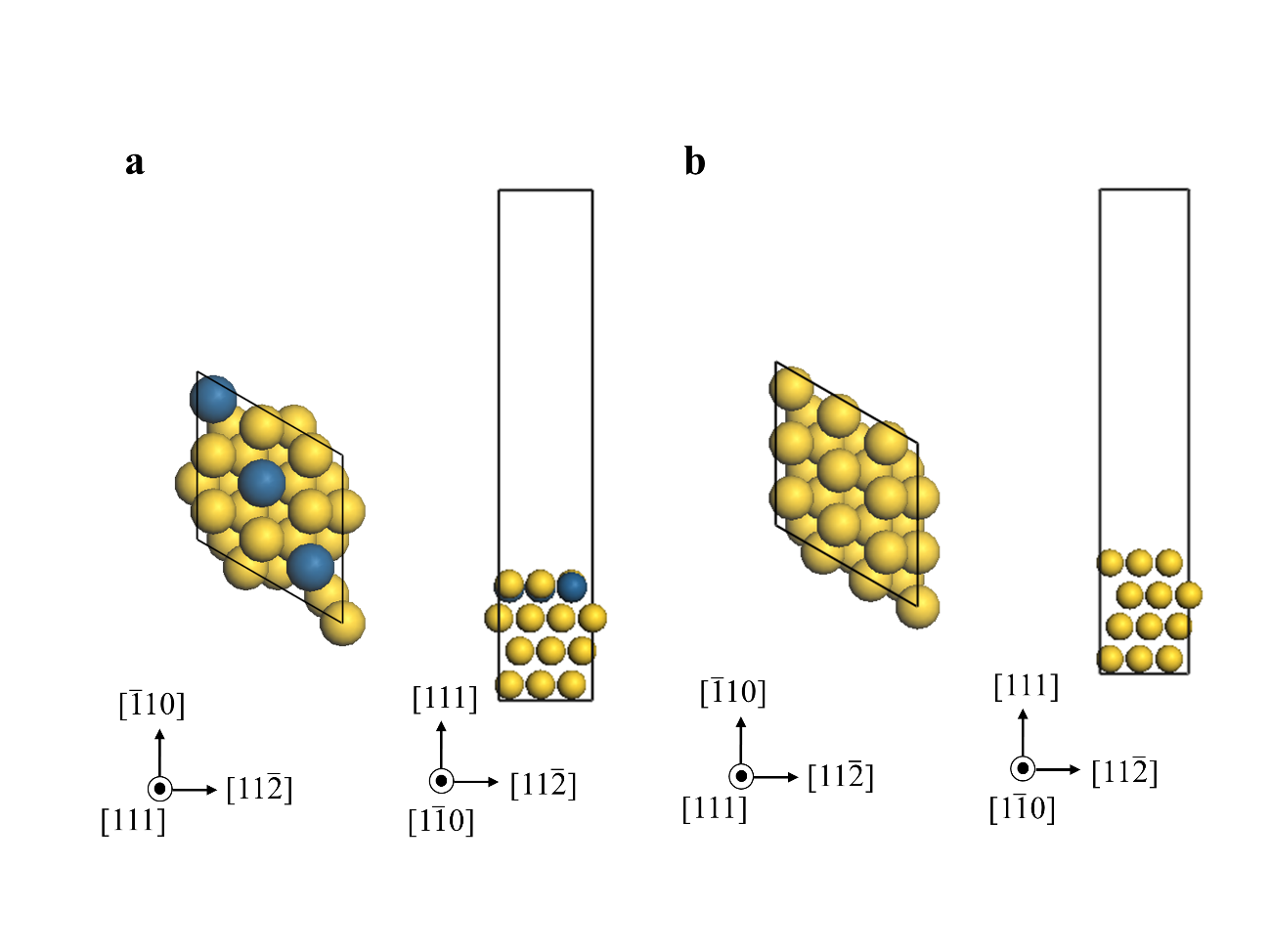


**Supplemental Figure 4. Simulation models used for first-principles calculation**. **a**, npAu-Pt model. **b**, npAu model. The models have a slab geometry with 4 atomic layers of 4×4 and a vacuum layer of 30 Å. The 5% compressive strain is loaded in the simulation models. The yellow and blue atoms are Au and Pt atoms, respectively.


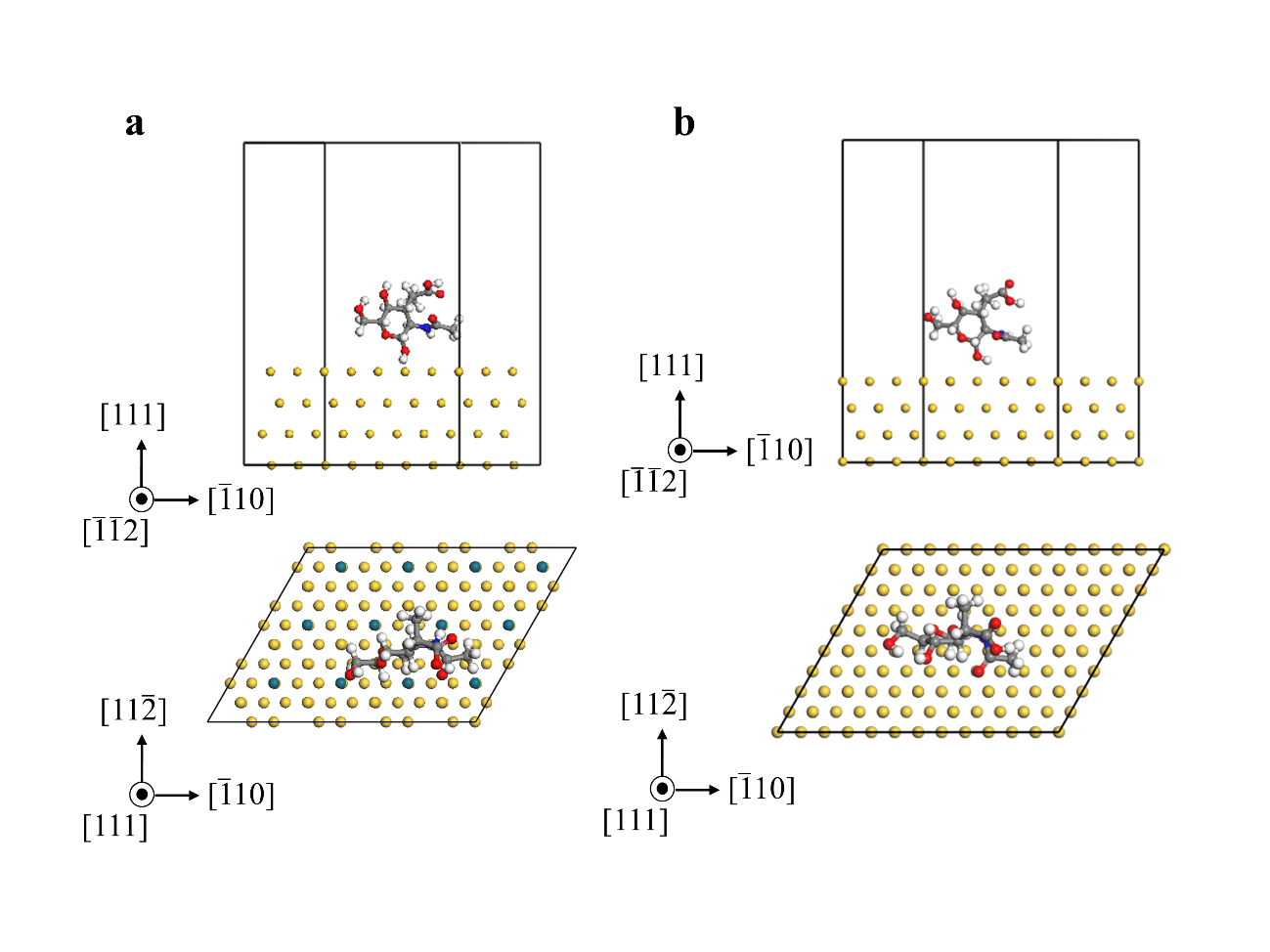


**Supplemental Figure 5. Simulation models used for adsorption of peptidoglycan on npAu and npAu-Pt models**. **a**, npAu-Pt model. **b**, npAu model. The models have a slab geometry with 4 atomic layers of 4×3and a vacuum layer of 15 Å. A 5% compressive strain is loaded in the simulation models. The white, gray, red, dark blue, yellow and blue atoms are H, C, O, N, Au and Pt atoms, respectively.

**Supplemental Table 1 Chemical compositions by EDX measurements.**

|  | Au (at.%) | Pt (at.%) | Ag (at.%) |
| --- | --- | --- | --- |
| npAu-Pt_0.5_ | 51.1 | 32.1 | 16.8 |
| npAu-Pt_0.1_ | 88.9 | 3.9 | 7.2 |
| npAu | 99.2 | - | 0.8 |
